# Prevalence of multimorbidity according to the deprivation level among the elderly in the Basque Country

**DOI:** 10.1186/1471-2458-13-918

**Published:** 2013-10-03

**Authors:** Juan F Orueta, Roberto Nuño-Solinís, Arturo García-Alvarez, Edurne Alonso-Morán

**Affiliations:** 1 O+berri, Basque Institute for Healthcare Innovation, Plaza Asua 1, 48150 Sondika, Spain; 2 Osakidetza, Basque Health Service, Centro de Salud de Astrabudua, Mezo 35, 48950 Erandio, Spain; 3 Kronikgune, Centro de Investigación en Cronicidad, María Diaz de Haro, nº 58 – 60 1ª pl, 48010 Bilbao, Spain

**Keywords:** Multimorbidity, Chronic diseases, Elderly, Aged, 80 and over, Inequalities

## Abstract

**Background:**

Multimorbidity is clearly a major challenge for healthcare systems. However, currently, its magnitude and impact on healthcare expenditures is still not well known. The objective of this paper is to present an overview of the prevalence of multimorbidity by deprivation level in the elderly population of the Basque Country.

**Methods:**

We conducted a cross-sectional analysis that included all the inhabitants of the Basque Country aged 65 years and over (N = 452,698). This was based on data from primary care electronic medical records, hospital admissions, and outpatient care databases, for a 4-year period. The health problems of the patients were identified from their diagnoses and prescriptions. Multimorbidity was defined as the presence of two or more chronic diseases out of a list of 47 of the most important and common chronic conditions consistent with the literature. In addition, we explored socio-economic and demographic variables such as age, sex, and deprivation level.

**Results:**

Multimorbidity was found in 66.13% of the population aged 65 and over and increases with age until 80 years. The prevalence of multimorbidity was higher in deprived (69.94%) than better-off (60.22%) areas. This pattern of differences between the most and least disadvantaged areas was observed in all age groups and more marked in female (70.96-59.78%) than in male (68.54-60.86%) populations. In almost all diseases studied (43 out of 47), 90% of patients had been diagnosed with at least one other illness. It was also frequent the coexistence of mental and physical health problems in the same person and the presence of multiple physical diseases is higher in patients with mental disease than in the rest of population (74.97% vs. 58.14%).

**Conclusion:**

Multimorbidity is very common among people over 65 years old in the Basque Country, particularly in unfavourable socioeconomic environments. Given the ageing population, multimorbidity and its consequences should be taken into account in healthcare policy, organization of care and medical research. Administrative health databases are readily available sources of a range of information that can be useful for such purposes.

## Background

Multimorbidity is a common problem in aged populations and has a wide range of individual and societal consequences. The costs of caring for chronic patients increase dramatically with the number of comorbidities. Given the ageing population and the implications of multimorbidity for patients, their families and caregivers, and for health systems and societies, policy makers, administrators, clinicians and researchers need to explore innovative approaches, in order to ensure that high quality care is provided [1,2].

The coexistence of multiple diseases in a single patient is so common that multimorbidity is now considered the most prevalent chronic health condition [3], and a recent systematic review of 41 published studies worldwide reported a rate of multimorbidity of between 55 and 98% in people aged 65 and older [4].

Certain common factors have been determined in relation to multimorbidity, such as age and unfavourable socio-economic conditions [5,6]. However, there are no universally accepted definitions for chronicity or multimorbidity [7] and many studies are based on a list of diseases the criteria for which have not been clearly explained [8]. Further, the use of different sources of information may lead to different results in terms of prevalence. These sources include surveys, diagnoses from administrative databases, prescriptions or primary care medical records. In addition, most studies have included only certain segments of the population, such as healthcare service users [6,9], users of healthcare centres taking part in certain programmes [10], or those whose data meet certain quality criteria [5,9,11-13]. Hence, it is difficult to establish whether the dissimilar values for the prevalence of multimorbidity that have been published are due to differences in the studied populations or to variations in the methodology used.

We sought to extend what is known about multimorbidity and, to that end, we designed a study employing a huge administrative database that contains information covering almost the entire population of the Basque Country. Taking advantage of and adapting a methodology already described in the literature [10], we developed a list of 47 diseases and defined specific criteria in order to consider a disease active in a given patient. The objective of this study was to assess the prevalence of multimorbidity in the population above 65 years old, and to explore differences as a function of sex, age and deprivation level.

## Methods

### Ethics statement

This study was approved by the Ethics Committee of the Basque Country.

We used databases that employ an opaque identifier to ensure patient confidentiality.

### Study population

The study population included every person aged 65 and over who was covered by public health insurance in the Basque Country on 31st August 2011 and who had been covered for at least 6 months in the previous year, regardless of whether or not they had made any contact with or use of the Basque Health Service (N = 452,698). Hence, our dataset is not a representative sample but rather we observe all the elderly inhabitants served by our health service, which corresponds to almost the entire elderly population in the Basque Country.

### Scope of the dataset

Data from the present study are derived from the database set up by the Population Stratification Programme (PREST) of the Basque Health Service. Although this programme began in 2010 with the aim of classifying all inhabitants of the autonomous region in terms of their future healthcare needs, the database contains information collected since 2007. It combines several different sources of information (primary and specialized health care registers and census data) and, from these, we obtained data on the following variables: diagnoses (primary care, specialist care, and hospitalisation), prescriptions and procedures. A more detailed description of these variables is available in a previous publication [14].

### Clinical information

In the Basque Health Service, diagnoses of hospital discharges, visits to specialists, and primary medical records are coded according to the ICD-9-CM system [15], while the Anatomical Therapeutic Chemical (ATC) [16] coding system is used for drugs prescribed by primary care doctors. With this information, inhabitants in the Basque Country are classified annually using Adjusted Clinical Groups (ACGs), a case mix system –originally developed by Starfield et al. [17] at Johns Hopkins University- that enables health problems to be identified from diagnoses and prescriptions and individuals to be categorized into a hundred groups according to their healthcare needs and its costs. Though there could be omissions or errors in doctors’ notes in the medical records, it is considered that they provide high-quality information. Notably, in a previous study, it was found the rates of chronic diseases estimated using data in administrative databases of the Basque Country are similar or (in many cases) higher than those obtained from population surveys [18].

### Definition of chronic conditions

With the aim of describing the prevalence of chronic diseases and multimorbidity, we adopted a list of 47 pathologies, defined by consensus among the research team. This task was based on adapting two pre-existing lists, published by other authors: the 40 diseases selected by Barnett el al. [10] and the conditions considered to be chronic in the ACG Technical Reference Guide [19].

Barnett and others [10] drew up a list of diseases based on their impact on patients in terms of need for chronic treatment, impaired function, reduced quality of life and risk of future morbidity or mortality. They included pathologies from a previous systematic review [8], the ones contained in the Quality and Outcomes Framework (QOF) of the UK General Practice contract and a selection of conditions considered important for health service planning by NHS Scotland. According to the characteristics of each disease, they employed different criteria: the presence of specific Read codes in the patient medical records, prescription of medications to treat the condition, or a combination of diagnoses and prescriptions. To avoid including inactive health problems, in some situations they established a period for diagnoses or prescriptions to be considered.

The ACG system identifies patients suffering specific health problems, by means of ICD-9-CM codes of diagnoses registered or medications prescribed. Based on the clinical characteristics of the condition, diagnoses are also classified in 264 Expanded Diagnosis Clusters (EDCs) and drugs into 69 Rx-defined Morbidity Groups (Rx-MGs). In this case-mix system, a chronic condition is defined as an alteration that is likely to last longer than 12 months and to have a negative impact on health or functional status, and it includes 127 EDCs.

For the purpose of this study, we adapted the list of Barnett et al. to our clinical data, i.e., we identified the conditions from EDCs, Rx-MGs, or ICD-9-CM or ATC codes, instead of British National Formulary or Read Codes. We also added to this selection a group of 10 relevant health problems from chronic EDCs. Subsequently, we omitted three pathologies: on the one hand, “painful condition”, because this category contains a very heterogeneous cluster of symptoms and diseases and, on the other, “attention deficit disorder” and “psychoactive substance misuse”, due to these diagnoses being very uncommon in the age group under study. Therefore, our definitive list was composed of 47 morbidities.

Though in most cases it is considered that a person has a chronic disease because it has been assigned the corresponding diagnosis (for example, congestive heart failure), for some illnesses other criteria were applied: diagnosis or prescription of specific medications (e.g., for diabetes mellitus and Parkinson’s); repeated diagnosis over several years (low back pain); any history of the diagnosis together with prescription of specific drugs in the previous year (asthma and epilepsy); diagnosis in the previous year or repeated prescriptions over several months (depression and anxiety); or repeated prescriptions to treat the given health problem (treated dyspepsia). A detailed description of the methods employed can be found in the Additional file 1.

In this study, we considered multimorbidity to be the co-occurrence of two or more health problems in the same person, this being the definition most widely used in the literature [20,21]. Nevertheless, given that there is not a clear consensus on the minimum number of illnesses a person must have to apply the term multimorbidity [1,7,22], we also identified the people in our population with three or more health problems, this being the criterion applied by some other authors [23].

### Socio-demographic information

Demographic variables were used (age on the final day of the study period and sex), along with the geographical deprivation index and chronic diseases. The deprivation index based on census tract was used as the social indicator. A tract is the smallest geographical unit into which population census data can be broken down, and these are created according to population size, as well as geographical and urban criteria. While the number of inhabitants in each tract varies, the median is around 1,200 per tract. As the tracts are so small, they tend to be quite homogenous with respect to the type of dwellings. The deprivation index provides a measure of the socio-economic characteristics of census tracts and is constructed from the following variables: percentage of manual workers, unemployment, temporary employment, and low educational attainment in the population (people who are illiterate or have not completed primary education), both overall and also specifically among young people (inhabitants between 16–29 years of age) [24]. Although this index is not specific to the elderly, it provides a measure of the level of access to material and social resources in a community and has been shown to be correlated with general rates of mortality. In this study, we categorised individuals into quintiles by the deprivation index score, where 1 corresponds to the least and 5 the most deprived.

### Statistical analysis

Data are described using means, frequencies, contingency tables and graphs. Non-parametric tests were used to compare the mean number of illnesses: the Wilcoxon Mann–Whitney test, for assessing differences between the sexes, and the Kruskal-Wallis test, for differences between levels of deprivation, and age groups, both overall and stratified by sex. In addition, the chi-square test was applied to explore differences in the percentage of the population found to have multimorbidity. All this analysis was performed using SAS version 9.2 (SAS Institute, Cary, NC).

## Results

In the Basque Country, 452,698 inhabitants are over 65 years and 145,780 over 80 years. This represents, respectively, 20.0 and 6.4% of the total population. As would be expected, these percentages are higher in women (22.6 and 8.2%) than in men (17.3 and 4.6%).

Table 1 presents the distribution of the population by sex and age groups. The average number of chronic diseases per person in the total elderly population was found to be 2.65. This value increases with age up to 80 years and is slightly higher in men, but differences between sexes are not statistically significant (p = 0.682). However, the disparities are larger in the female population, women living in the more deprived neighbourhoods having the highest mean number of pathologies.

**Table 1:** Mean numbers of chronic diseases and percentage of patients with comorbidity in patient groups according to sex, age and deprivation index quintile

	**No. of people**	**Average number of chronic diseases per patient. Mean (SD)**					
			**Deprivation index**				
		**Overall**	**1**	**2**	**3**	**4**	**5**
Population	452,698	2.65 (2.15)	2.37 (2.06)	2.60 (2.10)	2.72 (2.15)	2.73 (2.16)	2.87 (2.23)
Men							
Age group (years)							
65-69	57,267	2.03 (1.80)	1.82 (1.74)	1.99 (1.74)	2.11 (1.85)	2.10 (1.81)	2.18 (1.88)
70-74	41,649	2.49 (2.00)	2.26 (1.92)	2.46 (1.97)	2.58 (2.04)	2.55 (2.02)	2.59 (2.06)
75-79	42,016	2.98 (2.22)	2.74 (2.18)	2.93 (2.19)	3.05 (2.22)	3.06 (2.25)	3.11 (2.26)
80-84	30,110	3.34 (2.41)	3.10 (2.32)	3.37 (2.39)	3.45 (2.40)	3.35 (2.41)	3.44 (2.50)
85+	21,516	3.18 (2.57)	2.92 (2.49)	3.19 (2.51)	3.25 (2.60)	3.29 (2.61)	3.29 (2.64)
All Men	192,558	2.67 (2.19)	2.42 (2.12)	2.65 (2.16)	2.75 (2.21)	2.74 (2.21)	2.81 (2.26)
Women							
Age group (years)							
65-69	62,315	1.97 (1.73)	1.67 (1.61)	1.91 (1.70)	2.04 (1.75)	2.05 (1.75)	2.24 (1.83)
70-74	48,447	2.39 (1.91)	2.07 (1.80)	2.28 (1.84)	2.45 (1.91)	2.49 (1.92)	2.66 (2.03)
75-79	55,224	2.85 (2.10)	2.53 (2.01)	2.73 (2.04)	2.90 (2.09)	2.94 (2.12)	3.13 (2.19)
80-84	45,364	3.20 (2.25)	2.89 (2.19)	3.11 (2.19)	3.27 (2.22)	3.29 (2.26)	3.48 (2.36)
85+	48,790	2.99 (2.34)	2.72 (2.26)	2.96 (2.29)	3.07 (2.35)	3.06 (2.34)	3.22 (2.45)
All Women	260,140	2.64 (2.11)	2.34 (2.03)	2.56 (2.06)	2.70 (2.11)	2.72 (2.12)	2.91 (2.21)

Differences statistically significant among Deprivation Index groups (P < 0.0001).

**Figure 1:**
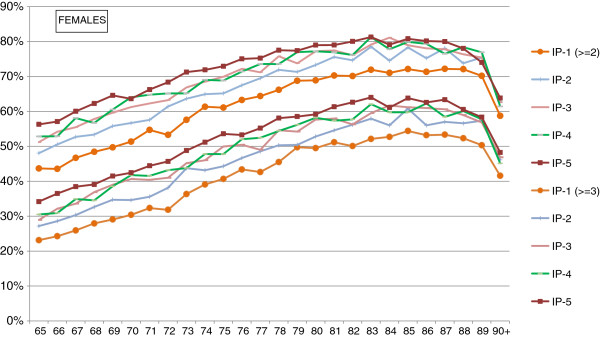
Percentage of women with multimorbidity (at least 2 and at least 3 chronic conditions) by deprivation index.

**Figure 2:**
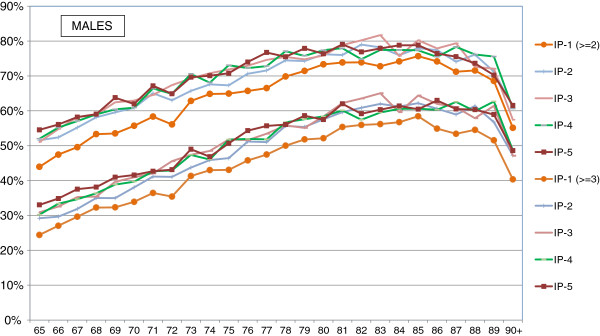
**Percentage of men with multimorbidity (at least 2 and at least 3 chronic conditions) by deprivation index.** Note: DI5 corresponds to the most deprived population.

More than half of over 65-year-olds and more than 75% of the population between 80 and 84 years old were classified as having multimorbidity, because they had at least two chronic health problems (Table 2), and the differences between sexes are not significant (p = 0.521). The coexistence of even more chronic conditions in the same person is also common, three or more pathologies being found in 30% of the population at 65 and 60% at 85 years of age. Again, the pattern of differences between the groups with the most and least favourable socio-economic status varied between the sexes. While women showed larger disparities and there was a strong gradient between these groups (Figure 1), in men discrepancies were less apparent: the lowest prevalences of multimorbidity were observed in the least disadvantaged, but there were relatively small differences between the other groups (Figure 2).

**Table 2:** Population with multimorbidity (two or more chronic diseases) by age, sex and deprivation index quintile

**All**			**Deprivation index**				
		**All**	**1**	**2**	**3**	**4**	**5**
		**66.11% (65.97-66.25)**	**60.22% (59.91-60.54)**	**65.33% (65.03-65.64)**	**67.63% (67.33-67.94)**	**67.86% (67.56-68.16)**	**69.94% (69.64-70.25)**
**Sex**	**Age group (years)**						
**Male**	**65-69**	54.94% (54.53-55.35)	49.18% (48.30-50.07)	54.93% (54.04-55.83)	56.69% (55.78-57.59)	56.55% (55.65-57.45)	58.13% (57.16-59.10)
	**70-74**	64.69% (64.23-65.15)	59.38% (58.32-60.44)	64.41% (63.39-65.44)	66.80% (65.78-67.82)	66.10% (65.11-67.09)	66.74% (65.71-67.76)
	**75-79**	72.49% (72.07-72.92)	67.57% (66.53-68.61)	71.54% (70.57-72.50)	73.89% (72.95-74.83)	74.16% (73.26-75.06)	74.85% (73.92-75.77)
	**80-84**	76.77% (76.29-77.25)	73.59% (72.45-74.72)	77.04% (75.99-78.08)	78.48% (77.43-79.53)	77.00% (75.97-78.04)	77.69% (76.61-78.76)
	**85+**	70.74% (70.13-71.35)	67.13% (65.78-68.49)	71.60% (70.28-72.91)	71.34% (69.98-72.70)	72.18% (70.83-73.52)	71.84% (70.41-73.27)
	**All**	66.06% (65.85-66.27)	60.86% (60.37-61.34)	65.93% (65.46-66.40)	67.61% (67.14-68.07)	67.52% (67.06-67.98)	68.54% (68.06-69.01)
**Sex**	**Age group (years)**						
**Female**	**65-69**	53.55% (53.16-53.94)	46.12% (45.28-46.96)	51.87% (51.00-52.74)	55.35% (54.47-56.23)	55.93% (55.06-56.81)	59.84% (58.93-60.74)
	**70-74**	62.92% (62.49-63.35)	55.55% (54.55-56.54)	60.71% (59.73-61.68)	64.34% (63.38-65.31)	65.68% (64.75-66.61)	68.17% (67.25-69.10)
	**75-79**	70.99% (70.61-71.37)	64.71% (63.80-65.61)	69.04% (68.17-69.90)	72.55% (71.70-73.39)	72.80% (71.99-73.62)	75.59% (74.79-76.38)
	**80-84**	76.16% (75.77-76.55)	70.40% (69.48-71.32)	75.27% (74.39-76.15)	78.07% (77.20-78.94)	77.80% (76.94-78.66)	79.64% (78.81-80.47)
	**85+**	70.66% (70.25-71.06)	66.11% (65.25-66.96)	71.02% (70.15-71.90)	71.95% (71.04-72.87)	72.00% (71.07-72.93)	73.66% (72.73-74.60)
	**All**	66.15% (65.97-66.33)	59.78% (59.37-60.19)	64.89% (64.48-65.30)	67.65% (67.25-68.06)	68.12% (67.72-68.53)	70.96% (70.57-71.36)

Percentage (95% CI) Differences statistically significant among Deprivation Index groups (P < 0.0001).

Men and women also had dissimilar patterns of pathologies. Although arterial hypertension and diabetes were found to be highly prevalent in both sexes, in men there were high rates of prostatic hypertrophy, malignancies, respiratory and cardiac diseases, while degenerative bone and joint diseases and mental problems were more common in females. Table 3 also illustrates that multimorbidity is very common in patients with any of the studied diseases. In almost all of them (43 out of 47), 90% of the patients had been diagnosed with at least one other illness and, in one third of cases, half of the patients had at least another four health problems.

**Table 3:** Prevalence of chronic diseases and comorbidities, in the population aged 65 and over in the Basque Country

**Diseases**	**Men**	**Women**	**Both sexes**	**Only this chronic condition**	**At least one more**	**At least two more**	**At least three more**	**At least four more**
	**N (%)**	**N (%)**	**N (%)**	**N (%)**	**N (%)**	**N (%)**	**N (%)**	**N (%)**
Hypertension	117907 (53%)	156985 (55%)	274892 (54%)	39725 (14%)	235167 (86%)	173639 (63%)	115512 (42%)	71671 (26%)
Diabetes mellitus	41477 (19%)	43783 (15%)	85260 (17%)	4014 (5%)	81246 (95%)	66366 (78%)	47984 (56%)	31943 (37%)
Degenerative joint disease	17032 (8%)	44950 (16%)	61982 (12%)	3775 (6%)	58207 (94%)	48583 (78%)	35822 (58%)	23981 (39%)
Treated dyspepsia	20726 (9%)	38328 (13%)	59054 (12%)	1825 (3%)	57229 (97%)	49973 (85%)	38925 (66%)	27220 (46%)
Anxiety & other neurotic, stress-related & somatoform disorders	15047 (7%)	42496 (15%)	57543 (11%)	4785 (8%)	52758 (92%)	44513 (77%)	33452 (58%)	22900 (40%)
Atrial fibrillation	22726 (10%)	21150 (7%)	43876 (9%)	696 (2%)	43180 (98%)	39171 (89%)	32331 (74%)	24580 (56%)
Depression	9677 (4%)	33376 (12%)	43053 (8%)	1210 (3%)	41843 (97%)	37433 (87%)	29771 (69%)	21390 (50%)
Malignancies	23850 (11%)	18110 (6%)	41960 (8%)	3288 (8%)	38672 (92%)	31849 (76%)	23344 (56%)	15662 (37%)
Glaucoma	16609 (7%)	24265 (8%)	40874 (8%)	2436 (6%)	38438 (94%)	31913 (78%)	23233 (57%)	15429 (38%)
Osteoporosis	2057 (1%)	36559 (13%)	38616 (8%)	3440 (9%)	35176 (91%)	28577 (74%)	20645 (53%)	13791 (36%)
Cerebrovascular disease	16539 (7%)	18155 (6%)	34694 (7%)	1200 (3%)	33494 (97%)	29883 (86%)	24167 (70%)	17792 (51%)
Prostatic hypertrophy	33548 (15%)	---	33548 (7%)	2742 (8%)	30840 (92%)	25092 (75%)	18234 (54%)	12247 (37%)
Emphysema, chronic bronchitis, chronic obstructive pulmonary disease	23032 (10%)	10359 (4%)	33391 (7%)	1576 (5%)	31815 (95%)	28036 (84%)	22632 (68%)	16888 (51%)
Ischemic heart disease	21019 (9%)	11185 (4%)	32204 (6%)	365 (1%)	31839 (99%)	28353 (88%)	22650 (70%)	16671 (52%)
Chronic heart disease, others	16661 (7%)	12997 (5%)	29658 (6%)	665 (2%)	28993 (98%)	26786 (90%)	22963 (77%)	18143 (61%)
Hypothyroidism	4108 (2%)	23897 (8%)	28005 (5%)	2227 (8%)	25778 (92%)	20904 (75%)	15138 (54%)	10139 (36%)
Low back pain	8327 (4%)	18224 (6%)	26551 (5%)	1457 (5%)	25094 (95%)	21463 (81%)	16312 (61%)	11176 (42%)
Dementia	7780 (3%)	17134 (6%)	24914 (5%)	1518 (6%)	23396 (94%)	20154 (81%)	15764 (63%)	11516 (46%)
Chronic kidney disease	10430 (5%)	10367 (4%)	20797 (4%)	191 (1%)	20606 (99%)	19245 (93%)	16613 (80%)	13260 (64%)
Heart failure	8434 (4%)	10289 (4%)	18723 (4%)	67 (0%)	18656 (100%)	18201 (97%)	16966 (91%)	14796 (79%)
Deafness, hearing loss	7871 (4%)	10115 (4%)	17986 (4%)	2211 (12%)	15775 (88%)	13006 (72%)	9779 (54%)	6712 (37%)
Asthma (currently treated)	6129 (3%)	11241 (4%)	17370 (3%)	3406 (20%)	13964 (80%)	11729 (68%)	9204 (53%)	6615 (38%)
Blindness & low vision	7150 (3%)	10035 (3%)	17185 (3%)	1239 (7%)	15946 (93%)	13745 (80%)	10681 (62%)	7632 (44%)
Peripheral neuropathy, neuritis	5030 (2%)	9673 (3%)	14703 (3%)	791 (5%)	13912 (95%)	11931 (81%)	9275 (63%)	6630 (45%)
Diverticular disease of intestine	5162 (2%)	8534 (3%)	13696 (3%)	442 (3%)	13254 (97%)	12001 (88%)	9835 (72%)	7451 (54%)
Gout	10332 (5%)	2488 (1%)	12820 (3%)	564 (4%)	12256 (96%)	10542 (82%)	8125 (63%)	5876 (46%)
Rheumatoid arthritis and autoimmune and connective tissue diseases	3949 (2%)	7606 (3%)	11555 (2%)	629 (5%)	10926 (95%)	9454 (82%)	7347 (64%)	5226 (45%)
Parkinson’s disease	3941 (2%)	5204 (2%)	9145 (2%)	461 (5%)	8684 (95%)	7584 (83%)	5939 (65%)	4270 (47%)
Chromosomal anomalies or inherited metabolic disorders	4311 (2%)	3593 (1%)	7904 (2%)	595 (8%)	7309 (92%)	6027 (76%)	4440 (56%)	3059 (39%)
Chronic liver or pancreatic disease	4101 (2%)	3000 (1%)	7101 (1%)	227 (3%)	6874 (97%)	6243 (88%)	5201 (73%)	4092 (58%)
Peripheral vascular disease	4732 (2%)	1269 (0%)	6001 (1%)	104 (2%)	5897 (98%)	5525 (92%)	4771 (80%)	3865 (64%)
Treated constipation	2100 (1%)	3816 (1%)	5916 (1%)	108 (2%)	5808 (98%)	5362 (91%)	4560 (77%)	3536 (60%)
Paralysis or muscular dystrophy	2701 (1%)	2684 (1%)	5385 (1%)	183 (3%)	5202 (97%)	4841 (90%)	4220 (78%)	3418 (63%)
Schizophrenia, affective psychosis or bipolar disorder	1487 (1%)	2500 (1%)	3987 (1%)	304 (8%)	3683 (92%)	3163 (79%)	2530 (63%)	1905 (48%)
Bronchiectasis	1582 (1%)	1897 (1%)	3479 (1%)	5 (0%)	3474 (100%)	3265 (94%)	2868 (82%)	2280 (66%)
Disorders of the immune system	1327 (1%)	1899 (1%)	3226 (1%)	176 (5%)	3050 (95%)	2710 (84%)	2202 (68%)	1712 (53%)
Irritable bowel syndrome	834 (0%)	2200 (1%)	3034 (1%)	144 (5%)	2890 (95%)	2562 (84%)	2044 (67%)	1464 (48%)
Epilepsy (currently treated)	1395 (1%)	1372 (0%)	2767 (1%)	228 (8%)	2539 (92%)	2153 (78%)	1741 (63%)	1288 (47%)
Psoriasis or eczema	1533 (1%)	1186 (0%)	2719 (1%)	107 (4%)	2612 (96%)	2272 (84%)	1793 (66%)	1254 (46%)
Viral hepatitis	1110 (0%)	1456 (1%)	2566 (1%)	156 (6%)	2410 (94%)	2088 (81%)	1679 (65%)	1266 (49%)
Hematologic chronic disorders	1048 (0%)	1124 (0%)	2172 (0%)	124 (6%)	2048 (94%)	1856 (85%)	1626 (75%)	1321 (61%)
Alcohol problems	1764 (1%)	372 (0%)	2136 (0%)	109 (5%)	2027 (95%)	1796 (84%)	1472 (69%)	1141 (53%)
Inflammatory bowel disease	974 (0%)	1096 (0%)	2070 (0%)	124 (6%)	1946 (94%)	1673 (81%)	1303 (63%)	941 (45%)
Chronic sinusitis	531 (0%)	901 (0%)	1432 (0%)	126 (9%)	1306 (91%)	1105 (77%)	851 (59%)	600 (42%)
Intellectual disability	742 (0%)	490 (0%)	1232 (0%)	692 (56%)	540 (44%)	258 (21%)	179 (15%)	117 (9%)
Migraine	117 (0%)	652 (0%)	769 (0%)	72 (9%)	697 (91%)	569 (74%)	406 (53%)	259 (34%)
Transplant status	474 (0%)	275 (0%)	749 (0%)	14 (2%)	735 (98%)	691 (92%)	605 (81%)	477 (64%)

Table 4 compares the prevalence of physical multimorbidity (defined as the presence of two or more non-mental diseases) in patients with concomitant mental health problems and those without. These prevalences are higher among people with mental diseases in all age, gender and deprivation index groups, and such differences are slightly larger in the younger age groups and in those who suffer less deprivation.

**Table 4:** Percentage of population with mental health problems and physical multimorbidity

	**Percentage of population suffering from mental health problems**	**Physical multimorbidity**			
		**Population with mental health problems**		**Population without mental health problems**	
		**%**	**CI**	**%**	**CI**
**All**	22.50%	74.97%	(74.70-75.23)	58.14%	(57.98-58.30)
**Men**	14.47%	79.16%	(78.68-79.63)	61.28%	(61.04-61.51)
**Women**	28.45%	73.39%	(73.07-73.71)	55.36%	(55.13-55.59)
**Age group (years)**					
**65-69**	18.16%	63.27%	(62.63-63.92)	46.59%	(46.28-46.90)
**70-74**	19.48%	72.16%	(71.49-72.82)	56.71%	(56.35-57,07)
**75-79**	22.78%	78.87%	(78.33-79.41)	64.69%	(64.35-65.03)
**80-84**	26.95%	81.53%	(80.99-82.06)	69.08%	(68.69-69.46)
**85+**	28.60%	79.12%	(78.56-79.68)	60.90%	(60.48-61.33)
**Deprivation Index**					
**DI1**	21.16%	71.67%	(71.05-72.30)	51.67%	(51.31-52.03)
**DI2**	22.13%	73.29%	(72.69-73.90)	57.44%	(57.08-57.80)
**DI3**	22.69%	75.89%	(75.30-76.48)	59.92%	(59.55-60.29)
**DI4**	22.44%	75.75%	(75.17-76.33)	60.29%	(59.93-60.65)
**DI5**	24.25%	78.09%	(77.53-78.65)	62.04%	(61.67-62.41)

CI Confidence Interval (95%).

Physical multimorbidity was considered the concurrence of two or more chronic non-mental diseases in the same person.

They were considered as Mental health problems: Depression; Anxiety & other neurotic, stress related & somatoform disorders; Alcohol problems; Dementia; Schizophrenia, affective psychosis or bipolar disorder; Intellectual disability.

## Discussion

Multimorbidity is such a widespread phenomenon in the elderly population that, in fact, only a small proportion of chronically ill elders have just one chronic health problem, while it is very common for them to be diagnosed with a constellation of diseases. Overall, we found that the proportion of patients with multimorbidity increases with age and unfavourable social conditions. It is also frequent the coexistence of mental and physical health problems in the same person and the presence of multiple physical diseases is higher in populations with mental disease. Even though the pattern of the mean number of chronic illnesses per person in men and women are, to some extent, similar, there are notable differences between the sexes in the prevalence of specific diseases and the degree to which women and men are affected by social factors: inequalities between residents of the most and the least deprived areas are noticeably more prominent in females than males. Particularly, the groups of women who live in the least disadvantaged areas reach the same prevalences of multimorbidity eight years later than those in the most disadvantaged areas; in contrast, the difference is only four years among men.

Although our results are consistent with previous studies, it is difficult to make comparisons due to the scope of datasets and the different methodologies. Our study involved a count of illnesses to identify people with multimorbidity, which is the method most widely reported in the literature. However, studies differ in the number of chronic diseases considered, the definitions thereof, the populations analysed and the sources of information [1]. The definition of multimorbidity itself, in terms of the number of health problems present, has some limitations since it does not consider very relevant factors related to individual disease burden, such as the severity of the illnesses, their chronology, and the interaction between them, that is, whether they co-occur by chance, one causes another or they have common risk factors [25].

Other authors have described the prevalence of multimorbidity increasing with age up to a certain point where it plateaus. This general pattern was seen in the population of the Basque Country, but with two peculiarities: first, the plateau started at about 80 years old, a more advanced age than observed in other places [22] and, second, there was even a slight fall in the prevalence of multimorbidity around 90 years of age. Hence, comparing our results with those found by Barnett et al. in Scotland [10], we observe a similar mean number of diseases and prevalence of multimorbidity in groups between 65 and 84 years, but lower values in the Basque Country in those 85 years of age and over.

Differences between social groups in the prevalence of chronic illnesses and variations in risk factors between the sexes have been described by other authors. For example, inequalities in diabetes [26] and obesity [27] have been observed to be larger among women, while disparities in alcohol abuse [28] and smoking [27] are more marked in men. In relation to multimorbidity, this phenomenon has been less well documented and our study demonstrates that there are also different trends in men and women in this respect.

Our study differs from others in the literature in that, by including almost all the inhabitants in the Basque Country, it avoids the potential bias that could arise by only using a restricted sample of the population. Furthermore, we used four years of clinical data from different sources, which helps to overcome the shortcomings of analysing any given source in isolation [18]. Estimates of prevalences of chronic illnesses and multimorbidity obtained from administrative databases are affected by the length of the observation period [29]. In particular, it is known that in a considerable proportion of patients with the diagnosis of a serious chronic disease entered in their health record one year, this information does not appear again in the record the following year [30,31]. An excessively long observation period, however, may lead to the inclusion of diseases with a prolonged course but which are not active at the time of the study. On the other hand, medication records can provide a list of illnesses that are being treated and, in some cases, it may be more complete than that derived from diagnoses [32] but a single prescription might not reliably indicate the presence of a chronic illness. Attempting to avoid these problems in this study, we combined information from several different sources and employed a method designed to distinguish active from non-active diseases.

### Limitations

Nevertheless, our study has certain limitations. Firstly, administrative databases clearly only contain information about problems that people report to health services. Therefore, the prevalence of diseases only reflects attended morbidity; that is, we cannot detect diseases that are present but which patients and doctors are unaware of (a very common situation in certain chronic diseases).

Our observed prevalence is also influenced by other factors, such as accessibility to healthcare services and help-seeking behaviour of patients. Even though a public health insurance system providing universal coverage and financed through taxes is associated with fewer barriers to healthcare access than other models of care, the situation is not perfect. Previous studies have identified a relationship between socio-economic deprivation and use of health resources in Spain [33,34] and other countries [35,36] and, in most cases, the pattern has been pro-poor and pro-rich inequity in the use of primary and specialised care respectively. Though recognising that this could have influenced the recording of some health problems, we believe that it would not have significantly changed the differences in prevalence of chronic diseases observed between the groups.

On the other hand, it could be thought that the high prevalence of multimorbidity observed would be biased by the fact that patients affected by a chronic disease have regular contact with health services and, hence, are more likely to be diagnosed with other health problems. While our study cannot rule out this possibility, other authors have shown that elderly people and patients with complex health problems are precisely the groups in which doctors have the most difficulty in coding illnesses, and that this leads to an under recording of diseases among these groups [37].

### Implications of multimorbidity for health policy

Our results have potential implications for the implementation of healthcare policies and the organization of healthcare services. They support the view that an approach based on considering diseases separately has many limitations [38] and that it is necessary to consider the implications of multimorbidity for approaches to treatments [39,40], the evaluation of results, the organization of healthcare services, and the financial burden of managing illnesses [41].

People living in more deprived areas have a higher prevalence of health problems and a higher multimorbidity burden. This fact is often not taken into account when planning the distribution of healthcare resources across geographical areas [42]. The interrelationship between poverty and poor health is complex and the associated mechanisms are not fully understood. Nevertheless, it is recognised that the characteristics of a neighbourhood, in relation to the provision of services, environment, security and social influences over health-related habits, may affect its inhabitants and, in fact, it has even been proved that changing address can influence health [43]. Some of these factors may be susceptible to interventions from healthcare services, and should also be considered in healthcare services management.

The combination of chronic illnesses causes patients a greater degree of disability than would be expected from each disease in isolation, and it is associated with a lower quality of life, psychological distress, poorer health outcomes, and a greater risk of mortality [44]. Such patients have specific health care needs [38], require more complex clinical management and incur higher health care costs [44].

Multimorbidity is a very complex phenomenon and not all patients with multiple health problems have the same characteristics. Therefore, further research is required to identify the diverse subgroups of patients with multimorbidity, in order to implement specific patient-centred care programmes.

## Conclusion

Multimorbidity is very common among the people 65 years of age in the Basque Country and, in fact, very few patients with a chronic disease have not been diagnosed with at least one further chronic health problem. Although, for all age and sex groups, prevalence of multimorbidity is higher in unfavourable socioeconomic environments, the patterns are different in male and female populations, with the differences between the most and the least disadvantaged groups being more marked in women than in men. Given the ageing population, multimorbidity should be taken into account in healthcare policy, management and medical research.

## Abbreviations

ACG: Johns Hopkins adjusted clinical groups case-mix system; ATC: Anatomical therapeutic chemical classification system; EDC: Expanded diagnosis clusters; ICD-9-CM: International classification of diseases 9th revision, clinical modification, Spanish version; PREST: Population stratification program of the Basque health; Rx-MG: Rx-defined morbidity groups.

## Competing interests

The authors declare that they have no competing interests.

## Authors’ contributions

All authors participated in the design of the study. JFO performed the validation of databases. RNS and JFO wrote the draft of the manuscript. All authors participated in interpretation of the data, as well as critically reviewing the manuscript and approving the final version.

## Pre-publication history

The pre-publication history for this paper can be accessed here:

http://www.biomedcentral.com/1471-2458/13/918/prepub

## Supplementary Material

Additional file 1: Table S1 List of chronic morbidities and criteria employed. Click here for file
