# Supplementary material for: Prevalence of multimorbidity according to the deprivation level among the elderly in the Basque Country
Source: BMC Public Health. 2013 Oct 3;13:918. doi: 10.1186/1471-2458-13-918 (PMC3852493; doi:10.1186/1471-2458-13-918)
Supplement: Additional file 1: Table S1 — List of chronic morbidities and criteria employed. [file 1471-2458-13-918-S1.doc]

ADDITIONAL FILE

Additional file 1: Table S1: List of chronic morbidities and criteria employed

| **Chronic condition** | **Criteria** |
| --- | --- |
| Alcohol problems | Dx of Alcohol dependence syndrome (ICD9CM: 303*) ever recorded |
| Anxiety & other neurotic, stress related & somatoform disorders | Dx of Anxiety, neuroses (EDC-PSY01) in the last 12 months OR prescription of anxiolytics OR hypnotics and sedatives (ATC-codes: N05B OR N05C) during 3 or more months |
| Asthma (currently treated) | Dx of Asthma (EDC- ALL04 OR ALL05) ever recorded AND Prescription of drugs to treat Airway Hyperactivity (RxMG-RESx040) in the last 12 months. |
| Atrial fibrillation | Dx of Atrial fibrillation and flutter (ICD9CM: 427.3*) ever recorded |
| Blindness & low vision | Dx of Blindness, Retinal disorders, Diabetic retinopathy, Age-related macular degeneration (EDC-EYE02 OR EYE03 OR EYE13 OR EYE15) ever recorded. |
| Bronchiectasis | Dx Bronchiectasis (ICD9CM=494*) ever recorded |
| Cerebro-vascular disease | Dx of Cerebrovascular disease (EDC-NUR05) ever recorded |
| Chromosomal anomalies or Inherited metabolic disorders | Dx of Chromosomal anomalies, Inherited metabolic disorders (EDC-GTC01 OR GTC02) ever recorded |
| Chronic heart disease, others | Dx of Congenital heart disease, Cardiac valve disorders, Cardiomyopathy, Generalized atherosclerosis (EDC-CAR04 OR CAR06 OR CAR07 OR CAR10) ever recorded |
| Chronic kidney disease | Dx of Chronic renal failure, nephritis, nephrosis (EDC-REN01 OR REN04) ever recorded |
| Chronic liver or pancreatic disease | Dx of Chronic liver disease, Chronic pancreatitis (EDC-GAS05 OR GAS12) ever recorded |
| Chronic sinusitis | Dx Chronic sinusitis (ICD9CM=473*) ever recorded |
| Deafness, hearing loss | Dx Deafness, hearing loss (EDC-EAR08) ever recorded |
| Degenerative joint disease | Dx Degenerative joint disease (EDC-MUS03) ever recorded |
| Dementia | Dx Dementia and delirium (EDC-NUR11) ever recorded |
| Depression | Dx of Depression (EDC-PSY09) in the last 12 months OR prescription of Antidepressants (ATC codes N06A ANTIDEPRESSANTS) during at last 4 months in the last year |
| Diabetes Mellitus | Dx of Diabetes Mellitus (EDC-END06 OR END07 OR END08 OR END09) OR Prescription of drugs to treat Diabetes (RxMG-ENDx030 OR ENDx040) ever recorded |
| Disorders of the immune system | Dx Disorders of the immune system (EDC-ALL06) ever recorded |
| Diverticular disease of intestine | Dx Diverticular disease of colon (EDC-GAS10) ever recorded |
| Emphysema, chronic bronchitis, COPD | Dx of Emphysema, chronic bronchitis, COPD (EDC- RES04) ever recorded |
| Epilepsy (currently treated) | Dx of Epilepsy and recurrent seizures (ICD9CM: 345*) ever recorded AND prescription of drugs to treat Seizure Disorder (RxMG-NURx050) in the last year |
| Glaucoma | Dx of Glaucoma (EDC-EYE08) OR prescription of drugs to treat Glaucoma (RxMG-EYEx030) ever recorded |
| Gout | Dx Gout (EDC-RHU02) ever recorded |
| Heart failure | Dx Congestive heart failure (EDC-CAR05) ever recorded |
| Hematologic chronic disorders | Dx of Hemolytic anemia, Hemophilia, coagulation disorder (EDC-HEM01 OR HEM07) ever recorded |
| HIV, AIDS | Dx of HIV, AIDS (EDC-INF04) ever recorded |
| Hypertension | Dx of Hypertension (EDC-CAR14 OR CAR15) OR Prescription of drugs to treat High Blood Pressure (RxMG-CARx030) ever recorded |
| Hypotiroidism | Dx of Hypothyroidism (EDC-END04) OR Prescription of drugs to treat Thyroid Disorders (RxMG-ENDx050) ever recorded |
| Inflammatory bowel disease | Dx Inflammatory bowel disease (EDC-GAS02) ever recorded |
| Irritable bowel syndrome | Dx Irritable bowel syndrome (EDC- GAS09) ever recorded |
| Ischemic Heart Disease | Dx of Ischemic heart disease or acute myocardial infarction (EDC- CAR03 OR CAR12) ever recorded |
| Low back pain | Dx Low back pain (EDC-MUS14) repeated in at least 3 of the last 4 years |
| Malignancies | Dx of Malignacy (MEDC-MAL) ever recorded |
| Migraine | (Dx of Migraine (EDC-NUR22) ever recorded AND prescription of drugs to treat Migraine Headache (RxMG-NURx03) in the last 12 months) OR (more than 4 prescriptions of Antimigraine preparations (ATC: N02C) in the las year) |
| Osteoporosis | Dx Osteoporosis (EDC-END02) ever recorded |
| Paralysis or muscular dystrophy | Dx of Muscular dystrophy, Quadriplegia and paraplegia, Spinal cord injury/disorders, Other paralytic syndromes, Cerebral palsy (EDC-NUR09 OR NUR12 OR NUR16 OR NUR17 OR NUR18) ever recorded |
| Parkinson’s disease | Dx of Parkinson's disease (EDC-NUR06) OR Prescription of drugs to treat Parkinson's disease (RxMG-NURx040) ever recorded |
| Peripheral neuropathy, neuritis | Dx Peripheral neuropathy, neuritis (EDC-NUR03) ever recorded |
| Peripheral vascular disease | Dx Peripheral vascular disease (EDC-GSU11) ever recorded |
| Prostatic hypertrophy | Dx Prostatic hypertrophy (EDC-GUR04) ever recorded |
| Psoriasis or eczema | Dx of Psoriasis, dermatitis, eczema (EDC-SKN12 OR SKN02) ever recorded AND prescription during 3 or more months in the last year of “Antipsoriatics” or “Corticosteroids, dermatological preparations Or "Agents for dermatitis, excluding corticosteroids" (ATC: D05 OR DO7 OR D11AH ) |
| Rheumatoid arthritis and autoimmune and connective tissue diseases | Dx of Rheumatoid arthritis or Autoimmune and connective tissue diseases (EDC-RHU05 OR RHU01) ever recorded |
| Schizophrenia, affective psychosis or bipolar disorder | Dx of Schizophrenia and affective psychosis or Bipolar disorder (EDC-PSY07 OR PSY12) ever recorded |
| Transplant status | Dx Transplant status (EDC-ADM03 ) ever recorded |
| Treated constipation | Dx of constipation (EDC-GAS03) ever recorded AND more than 4 prescription of laxatives (ATC: A06) in the last year |
| Treated dyspepsia | Drugs for peptic ulcer and gastro-oesophageal reflux disease (ATC code: A02B) during 3 or more months, without prescription of antiinflammatory and antirheumatic products, non-steroids (ATC code: M01A) NOR Platelet aggregation inhibitors excl. heparin (ATC code: B01AC) in the same month. |
| Viral Hepatitis | Dx Viral hepatitis (ICD9CM=070*) ever recorded |

Dx: Diagnosis

EDC: Expanded Diagnosis Clusters

Rx-MGs: Rx-defined Morbidity Groups.

ICD9MC: Spanish version of the Electronic International Classification of Diseases, Ninth Revision, Clinical Modification.

ATC:Anatomical Therapeutic Chemical Classification System
